# Effects of *Nepeta cataria* and *Lactuca sativa* on Microbial Community Structure and Nitrogen–Phosphorus Biogeochemical Cycling in Aquaculture Water

**DOI:** 10.3390/biology15201860

**Published:** 2026-10-16

**Authors:** Kunjie Wu, Chengliang Hou, Chen Zhang, Tianyu Ren, Qiang Meng, Gaoliang Yuan, Chenxi Ju

**Affiliations:** School of Fisheries, Xinyang Agriculture and Forestry University, Xinyang 464000, China; 2003210006@xyafu.edu.cn (K.W.); 16691502240@163.com (C.H.); hnxyzc321@163.com (C.Z.); 16639770903@163.com (T.R.); 18939411686@163.com (Q.M.); glyuan6@outlook.com (G.Y.)

**Keywords:** *Nepeta cataria*, *Lactuca sativa*, aquaculture water, microbial community structure, nitrogen and phosphorus cycles

## Abstract

As the scale of aquaculture continues to expand, aquaculture waters are under increasing environmental pressure, particularly regarding total nitrogen (TN) and total phosphorus (TP). To address this challenge, this study evaluated the efficacy of *Nepeta cataria* and *Lactuca sativa* in removing nitrogen and phosphorus from aquaculture water and investigated the underlying microbial mechanisms. The experiment consisted of three groups: the *Nepeta cataria* group (NC) with *Nepeta cataria* planted, the *Lactuca sativa* group (HL) with *Lactuca sativa* planted, and the control group (CT) without any plants. The results indicated that, compared with the control group, both the *Nepeta cataria* group and the *Lactuca sativa* group significantly reduced the concentrations of TAN, nitrite, nitrate, TN, and TP in the aquaculture water (*p* < 0.01). Plant species exerted a significant regulatory effect on both planktonic and rhizosphere microorganisms. The phyla *Proteobacteria*, *Actinobacteriota*, and *Bacteroidota* were the core dominant phyla across all groups; hgcI_clade, *Rhodoluna*, and *Sediminibacterium* were the signature dominant genera in the plant groups’ planktonic water. The microbial community is highly coupled with nitrogen and phosphorus nutrients. *Nepeta cataria* and *Lactuca sativa* enhanced the nitrification–denitrification coupling process by enriching microbial communities with nitrogen and phosphorus transformation functions, thereby forming a synergistic purification model of “plant absorption–microbial transformation.” This study elucidated the regulatory mechanisms by which *Nepeta cataria* and *Lactuca sativa* influence microbial communities and nitrogen and phosphorus cycles in aquaculture water, providing a theoretical basis and technical reference for plant selection in aquaponics systems and the ecological purification of aquaculture water.

## Simple Summary

The rapid expansion of aquaculture has caused serious water pollution due to excessive nitrogen and phosphorus accumulation, which threatens aquaculture water environments. This study aimed to explore an eco-friendly water purification method by testing two common plants, catnip and lettuce, and analyzing how they improve polluted aquaculture water through microorganisms. The experimental results showed that both plants can effectively reduce various nitrogen and phosphorus pollutants in aquaculture water. These plants regulate the structure of water and root microbial communities, enrich beneficial microorganisms that transform nitrogen and phosphorus, and boost key nutrient conversion processes in water. The cooperation of plant nutrient absorption and microbial decomposition realizes efficient water purification. This research clarifies the ecological purification mechanism of the two plants, offering practical and theoretical support for selecting suitable plants to purify aquaculture water and build sustainable ecological aquaculture systems.

## 1. Introduction

China’s freshwater aquaculture output reached 36.7476 million tonnes in 2025 [1]. As the scale of aquaculture continues to expand, aquaculture waters face significant environmental pressures, with total nitrogen (TN) and total phosphorus (TP) being the primary factors contributing to exceedances in inland fishery waters [2]. As limiting nutrients for planktonic algae and aquatic plants in water bodies, excessive inputs of total nitrogen and total phosphorus can trigger eutrophication, leading to algal blooms or red tides [3]. Algal blooms or red tides disrupt aquatic ecological structures; massive algal proliferation blocks sunlight, causing the death of submerged plants; nocturnal oxygen consumption and the decomposition of algal remains further lead to water hypoxia, causing aquatic organisms such as fish and shrimp to suffocate. This results in a decline in biodiversity and the collapse of the water ecosystem’s self-purification capacity, which is also one of the primary causes of water eutrophication [4]. Therefore, the nitrogen and phosphorus content in aquaculture effluent is a key factor affecting the sustainable development of aquaculture, and the removal of nitrogen and phosphorus is a priority in the treatment of aquaculture effluent. Although traditional physical and chemical methods for nitrogen and phosphorus removal are effective, they suffer from high costs, high energy consumption, and the potential for secondary pollution. For example, drum-type microfiltration units are widely used to remove uneaten feed and feces from aquaculture effluent [5] and are relatively effective, but they are expensive; ozone can effectively remove ammonia nitrogen and nitrite from aquaculture effluent [6], but it can easily lead to air pollution or residual contamination in water bodies [7]. In contrast, plant–microbial synergistic remediation technology, as an environmentally friendly and low-cost ecological restoration method, has demonstrated great potential in the treatment of aquaculture effluent.

Research indicates that integrating aquaculture with plant cultivation—known as aquaponics—can effectively mitigate the negative impacts of effluent discharge and environmental pollution associated with traditional aquaculture models [8]. Aquaponic systems harbor diverse microbial communities with specific metabolic pathways [9]. These microorganisms play a key role in nutrient cycling and maintaining system stability [10,11], converting nitrogen and phosphorus nutrients from fish excreta and uneaten feed into forms that plants can utilize [12]. Plants grow by absorbing nutrients, thereby reducing the concentration of nutrients such as nitrogen and phosphorus in the system, improving water quality, and consequently benefiting fish farming [9]. At the same time, these microorganisms promote denitrification in the water through nitrogen cycling processes such as denitrification and anaerobic ammonium oxidation. However, current research on how the microbial community in aquaponics systems is shaped—particularly the roles played by different plants in shaping the microbial community structure—remains limited. This limits the generalizability of many research conclusions and restricts the widespread adoption of aquaponics systems [13]. *Lactuca sativa* is a fast-growing leafy vegetable that thrives on ammonium-based nutrients in aquaponic systems, making it an ideal choice for hydroponic cultivation [14]. Hendrik Monsees evaluated the growth of *Lactuca sativa* in decoupled aquaponic systems compared to conventional hydroponic systems and found no significant differences between the two groups [15]; in fact, the nutritional quality of the *Lactuca sativa* from decoupled systems was even superior to that of conventional hydroponic *Lactuca sativa* [16]. Although there is already a relatively thorough understanding of the effectiveness of *Lactuca sativa* in aquaponic systems, there remains a lack of knowledge regarding the role *Lactuca sativa* plays in shaping the microbial community structure within these systems.

As a traditional Chinese vegetable, *Nepeta cataria* contains numerous bioactive compounds, yet its application in aquaponic systems has not yet been reported [17]. As an edible traditional vegetable, *Nepeta cataria* produces abundant bioactive terpenoids. Beyond water purification functions in aquaponics, its harvested biomass can be directly used for human consumption. Its secondary metabolites also exhibit inhibitory effects against aquatic pathogenic bacteria, implying potential prospects as functional feed additives for aquaculture animals. Its multi-harvest growth trait may generate extra economic income for aquaponics systems, though feeding-relevant verification experiments remain to be implemented.

Based on the above theoretical framework, we hypothesized that *Nepeta cataria* and *Lactuca sativa* could reshape the rhizosphere and planktonic microbial community via root exudates, enrich functional microbes related to nitrogen–phosphorus transformation, and enhance nitrification–denitrification coupling processes, thereby improving aquaculture water quality. To test this hypothesis, we established a symbiotic experimental system comprising *Nepeta cataria*, *Lactuca sativa*, and *Micropterus salmoides* to analyze the effects of these two plants on nitrogen and phosphorus nutrient levels in the water. It further examined differences in microbial community composition across treatment groups at the phylum and genus levels, and employed redundancy analysis and other methods to reveal the correlations between microorganisms and nitrogen and phosphorus nutrient levels. The study aims to elucidate the ecological functional differences between *Nepeta cataria* and *Lactuca sativa* in aquaculture ponds, providing a scientific basis for optimizing plant configurations in aquaponics systems and mitigating nitrogen and phosphorus pollution in aquaculture water bodies.

## 2. Materials and Methods

### 2.1. Experimental Materials and Animals

**Figure 1:**
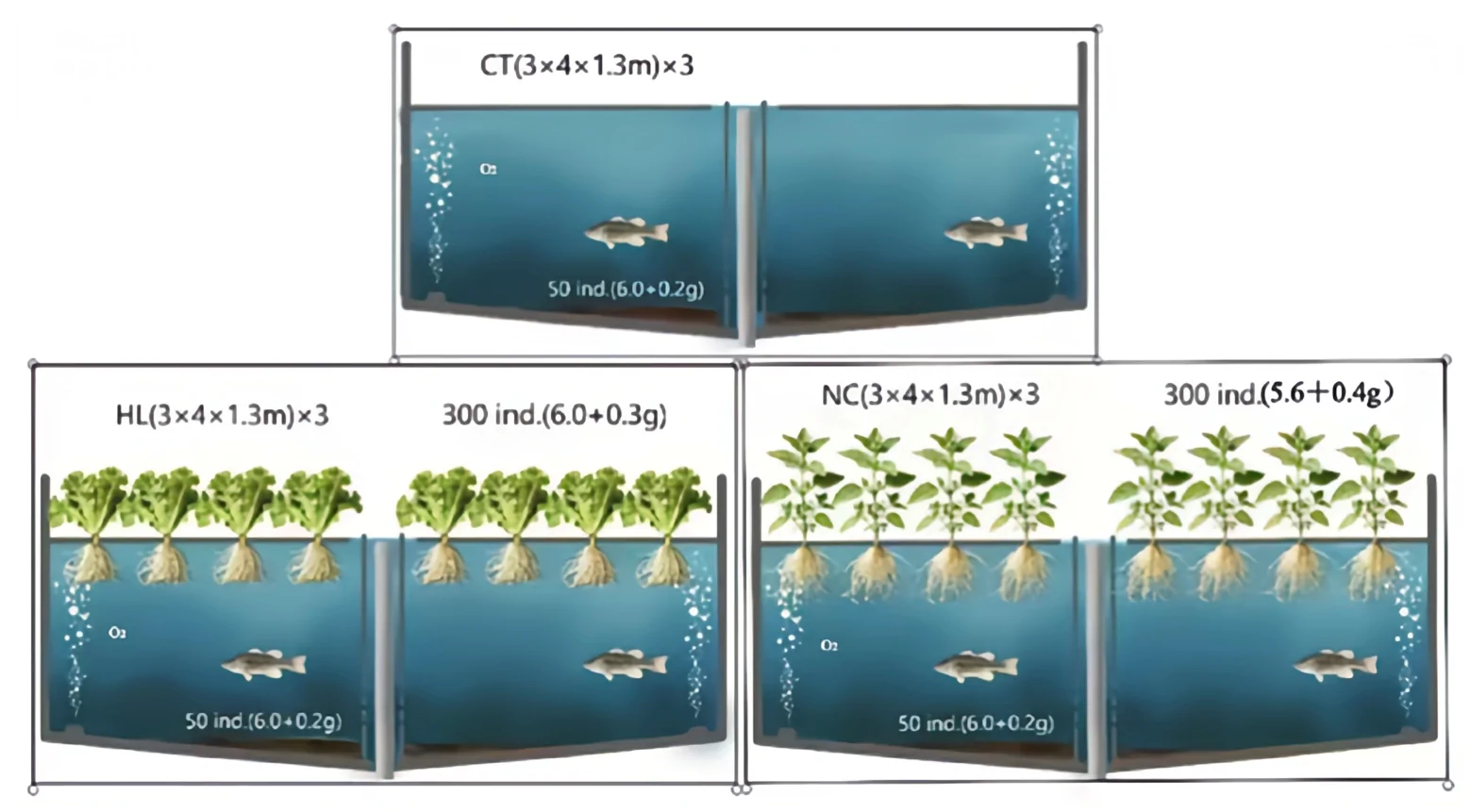
Grouping and stocking information of each experimental group.

The experiment was conducted from May to July 2024 at the on-campus research station of Xinyang Agricultural and Forestry College in Xinyang City, Henan Province (114.12408915474109, 32.164574957834546). The experiment consisted of three groups: the *Nepeta cataria* group (NC), the *Lactuca sativa* group (HL), and the No-plant group (CT, control group) (Figure 1). Each group had three replicates, totaling nine concrete tanks (length × width × depth: 3 × 4 × 1.3 m). Prior to the start of the experiment, a layer of approximately 5 cm of bottom sediment was spread on the pool bottom. After disinfection with bleaching powder, the pools were filled to a depth of 1 m. Air stones were placed at the four corners of each experimental pool for aeration. Polystyrene boards (3 cm × 60 cm × 120 cm) were laid across the water surface of all groups to ensure identical shading conditions, and a 1 m × 1 m feeding area was reserved in the centre. On 3 May 2024, a total of 450 disease-free and injury-free *Micropterus salmoides* of uniform size (6.0 ± 0.2 g) were weighed and randomly and evenly distributed into nine concrete tanks (fingerlings purchased from Wanghuating Fish Hatchery, Fangcheng County, Nanyang City, Henan Province). Electrical conductivity (EC) was measured daily. After the EC of the culture water reached 0.5 mS/cm (meeting the nutritional requirements for vegetable seedlings), seedlings of catnip and *Lactuca sativa* were cultivated (seedlings provided by Beijing Dingfeng Modern Agriculture Development Co., Ltd., Beijing, China). After the catnip and *Lactuca sativa* seedlings developed true leaves, transplanting was performed on 4 June 2024, which was defined as Day 0 of the experiment, for the *Nepeta cataria* group and *Lactuca sativa* group, respectively. The transplanting density was 40 plants/m^2^ for both *Nepeta cataria* seedlings (5.6 ± 0.4 g) and *Lactuca sativa* seedlings (6.0 ± 0.2 g). Holes (3 cm diameter) were drilled in the polystyrene boards for vegetable planting; the hole spacing was set to 15 cm to guarantee canopy light access, ventilation, and sufficient space for root development. Seedlings were secured in 3 cm-diameter planting cups, which were then inserted into the pre-drilled polystyrene boards.

### 2.2. Farming Management

Depending on the growth stage of the largemouth bass, the fish were fed Feed No. 1, No. 2, and No. 3 (Shenzhen Aohua Group Co., Ltd., Shenzhen, China), with crude protein contents of 45%. Feeding was conducted once in the morning (7:00–8:00) and once in the evening (18:00–19:00). During the early rearing phase, the feeding rate was 4% of the largemouth bass’s body weight; in the later phase, the feeding rate was reduced to 2%. The total daily feed intake for each concrete tank was recorded. No veterinary supplements or medications were used during the rearing process. Water was topped up regularly to a depth of 1 m, but no water changes were performed. The rearing period lasted 91 days.

### 2.3. Sample Collection and Analysis

#### 2.3.1. Water Sampling, Parameter Measurement and Statistical Analysis

Water samples were collected on 23 July 2024, with three replicates per group. Water sampling and the determination of physicochemical parameters were carried out according to the procedures described in the Water and Wastewater Monitoring and Analysis Methods. The following parameters were measured in the water: ammonia nitrogen (TAN) (Nessler’s reagent spectrophotometric method), nitrite nitrogen (N-(1-naphthyl)ethylenediamine photometric method), nitrate (UV spectrophotometric method), chemical oxygen demand (COD_Mn_) (acidic potassium permanganate method), and total phosphorus (TP) (stannous chloride reduction spectrophotometric method).

The data obtained from this study were analyzed using SPSS (version 22.0) and Excel 2019. A One-way analysis of variance (one-way ANOVA) followed by Tukey’s HSD test was conducted to evaluate the effects of different treatments. Artworks were made with Origin 2018.

#### 2.3.2. Microbial Sample Collection

All microbial samples were collected on 23 July 2024. Water microorganisms: Water samples were collected using a plexiglass sampler (2.5 L model) (Jukai Kechuang Co., Ltd., Weifang, China) at a depth of 20–30 cm in the center of the pond. The samples were filtered through a 0.22 μm sterile filter membrane (Millipore 0.22 μm, Merck Millipore, Darmstadt, Germany) using a vacuum filtration pump, transferred to 15 mL sterile centrifuge tubes, and stored at −80 °C for later use.

Rhizosphere microorganisms: Randomly selected root samples of *Nepeta cataria* tenuifolia and *Lactuca sativa* were suspended in PBS buffer (0.15 mol/L NaCl, 0.1 mol/L Na_2_EDTA, pH 8.0) and shaken thoroughly. The suspension was filtered through a 0.22 μm sterile filter membrane, then transferred to a 15 mL sterile centrifuge tube and stored at −80 °C for later use.

Sediment Microorganisms: Placed the samples directly into 15 mL sterile centrifuge tubes and stored immediately at −80 °C for analysis.

### 2.4. Sample DNA Extraction and Microbial Community Structure Analysis

#### 2.4.1. DNA Extraction and PCR Amplification

Following the manufacturer’s instructions, total genomic DNA from the microbial community was extracted using the E.Z.N.A.^®^ Soil DNA Kit (Omega Bio-Tek, Norcross, GA, USA). DNA quality was assessed via 1% agarose gel electrophoresis, and DNA concentration and purity were determined using a NanoDrop 2000 (Thermo Scientific, Waltham, MA, USA). The V3–V4 hypervariable region of the bacterial 16S rRNA gene was amplified using the specific primer set 338F (5′-ACTCCTACGGGAGGCAGCAG-3′) and 806R (5′-GGACTACHVGGGTWTCTAAT-3′) [18]. To distinguish different samples for high-throughput multiplex sequencing, barcodes were added to the 5′ end of reverse primers. PCR amplification was performed on an ABI GeneAmp^®^ 9700 thermal cycler using TransGen AP221-02 (TransStart FastPfu DNA Polymerase, TransGen Biotech Co., Ltd., Beijing, China).

The 20 μL PCR reaction system contained 4 μL of 5 × FastPfu buffer, 2 μL of 2.5 mM dNTP mixture, 0.8 μL forward primer (5 μM), 0.8 μL reverse primer (5 μM), 0.4 μL TransStart FastPfu DNA polymerase, 0.2 μL BSA, and 10 ng template genomic DNA, with ddH_2_O used to adjust the final volume to 20 μL. The thermal cycling program was set as follows: an initial denaturation at 95 °C for 3 min (1 cycle), followed by 27 amplification cycles of 95 °C for 30 s, 55 °C for 30 s, and 72 °C for 45 s, and a final extension at 72 °C for 10 min; the reaction was then maintained at 10 °C until termination. A no-template negative control was included in each PCR batch to monitor reagent contamination, and each sample was amplified in three technical replicates to minimize PCR amplification bias.

Subsequently, PCR amplicons were separated and recovered from a 2% agarose gel, purified using the DNA gel recovery and purification kit (PCR Clean-Up Kit, Yuhua, China), and accurately quantified with a Qubit 4.0 (Thermo Fisher Scientific, USA). All qualified amplicons were mixed at equimolar concentrations for subsequent library construction and Illumina high-throughput sequencing.

#### 2.4.2. Library Construction and Sequencing

The purified PCR products were used for library construction with the NEXTFLEX Rapid DNA-Seq Kit. The steps included: (1) adapter ligation; (2) removal of adapter-ligated fragments by magnetic bead selection; (3) enrichment of library templates by PCR amplification; (4) purification of PCR products using magnetic beads to obtain the final sequencing library. Sequencing was performed on the Illumina NextSeq 2000 platform (Shanghai Meiji Biotechnology Co., Ltd., Shanghai, China). Raw data were deposited in the NCBI SRA database.

#### 2.4.3. Data Quality Control and Assembly

After quality control of the raw sequencing data using fastp (https://github.com/OpenGene/fastp (accessed on 20 August 2024), version 0.19.6), assembly was performed using FLASH (https://github.com/OpenGene/fastp (accessed on 20 August 2024), version 1.2.11). The process primarily included: (1) Filtering out bases with a quality score below 20 at the end of reads; setting a 50 bp window; if the average quality score within the window was below 20, trimming the bases from the end of the window; filtering out reads shorter than 50 bp after quality control; and removing reads containing N bases; (2) Merging paired-end (PE) reads into a single sequence based on their overlap relationships, with a minimum overlap length of 10 bp; (3) The maximum allowed mismatch rate in the overlap region of the assembled sequences was 0.2; sequences that do not meet this criterion were filtered out; (4) Samples were distinguished based on the barcodes and primers at the beginning and end of the sequences, and the sequence orientation was adjusted; the allowed number of barcode mismatches was 0, and the maximum number of primer mismatches was 2.

#### 2.4.4. OTU Clustering and Species Annotation

Using the UPARSE v7.1 software (http://drive5.com/uparse/ (accessed on 20 August 2024)), the quality-controlled and assembled sequences were clustered into Operational Taxonomic Units (OTUs) based on 97% similarity, and chimeric sequences were removed. At the same time, all sequences annotated to chloroplasts and mitochondria were eliminated from all samples (if a sample contained chloroplast or mitochondrial contamination, it was recommended to exclude it). To minimize the impact of sequencing depth on subsequent alpha and beta diversity analyses, the number of sequences for all samples was normalized to 20,000 (sequence normalization is recommended); after normalization, the average sequence coverage (Good’s coverage) for each sample remained as high as 99.09%. The RDP Classifier (http://sourceforge.net/projects/rdp-classifier/ (accessed on 20 August 2024)) was used against the Silva 16S rRNA gene database (v138) for OTU taxonomic annotation, with a confidence threshold set at 70%. Community composition for each sample was analyzed at different taxonomic levels, and 16S functional prediction analysis was performed using the PICRUSt2 (version 2.2.0) software.

### 2.5. Statistical Analysis

All data analyses were performed on the Meiji BioCloud Platform (https://cloud.majorbio.com (accessed on 20 August 2024)), as follows: Mothur software (version v.1.30.2, http://www.mothur.org/wiki/Calculators (accessed on 20 August 2024)) was used to calculate alpha diversity metrics, including the Chao1 and Shannon indices. The Wilcoxon rank-sum test was applied to evaluate intergroup differences in alpha diversity; PCoA (Principal Coordinate Analysis) was used to assess the similarity of microbial community structures among samples, and the non-parametric PERMANOVA test was performed to detect intergroup differences in microbial community structures. LEfSe (Linear Discriminant Analysis Effect Size) analysis (http://huttenhower.sph.harvard.edu/LEfSe (accessed on 20 August 2024)) (LDA > 2, *p* < 0.05) was used to identify differentially abundant bacterial taxa from phylum to genus levels among groups. Distance-based redundancy analysis (db-RDA) was used to explore the influences of environmental factors on bacterial community structure in aquaponics systems and aquaculture water. Correlation analysis was conducted based on Spearman’s correlation coefficient (|*r*| > 0.6, *p* < 0.05). The Benjamini–Hochberg false discovery rate (FDR) method was used to adjust raw *p*-values for multiple testing. Correlations with FDR-adjusted *p* < 0.05 were considered statistically significant.

## 3. Results

### 3.1. Differences in the Physicochemical Properties of the Aquaculture Water

**Figure 2:**
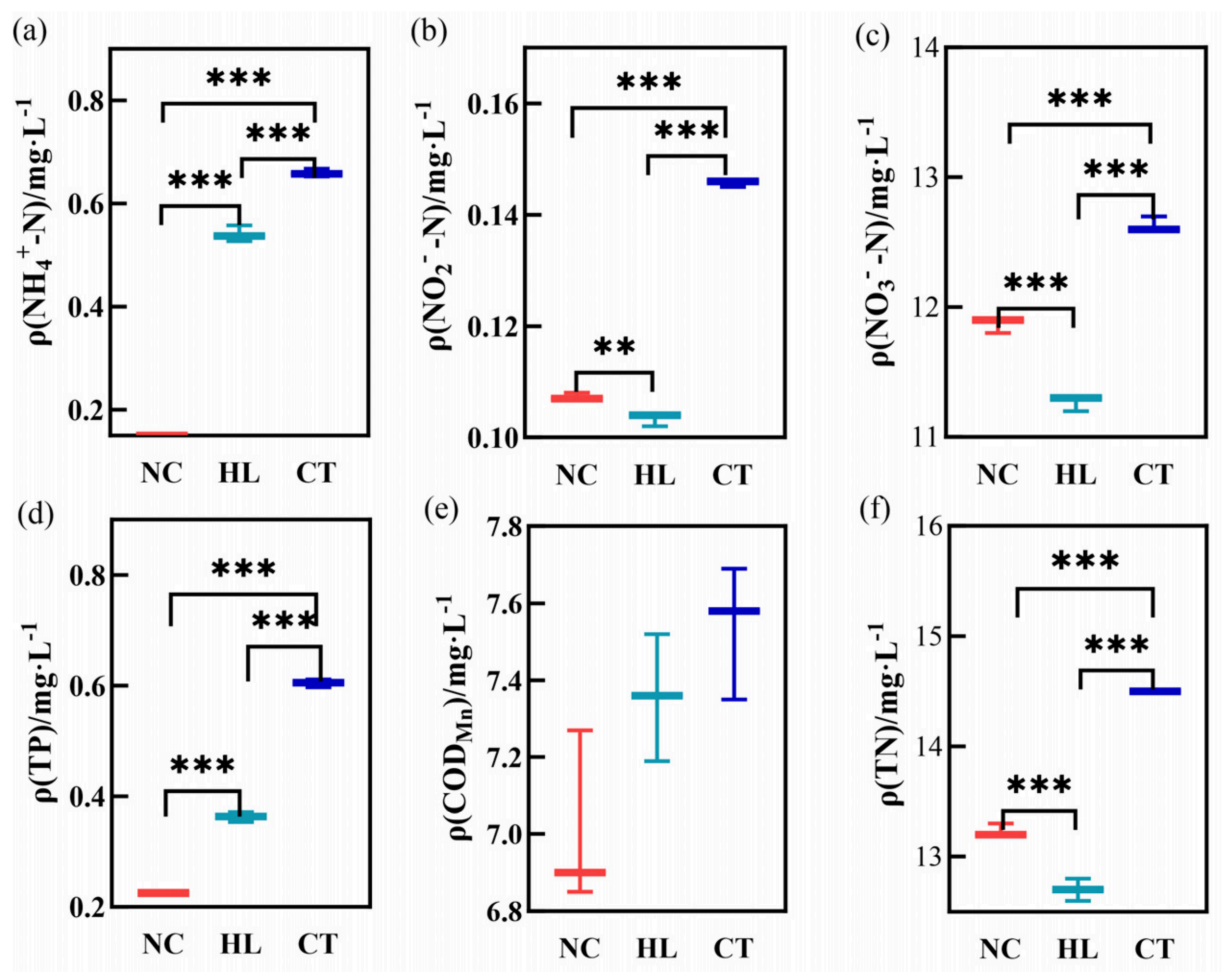
Concentrations of nitrogen and phosphorus nutrients under different treatments. (**a**) TAN; (**b**) nitrite; (**c**) nitrate; (**d**) TP; (**e**) CODMn; (**f**) TN. ** (0.001 ≤ *p* < 0.01), *** (*p* < 0.001).

As shown in Figure 2, except for COD_Mn_, significant differences were observed in environmental factors among groups (*p* < 0.01). NC had the lowest TAN (Figure 2a) and TP (Figure 2d), while CT had the highest; HL had the lowest nitrite (Figure 2b), nitrate (Figure 2c), and TN (Figure 2f), while CT had the highest.

### 3.2. Variations in Microbial Community Structure of the Aquaculture Water

**Figure 3:**
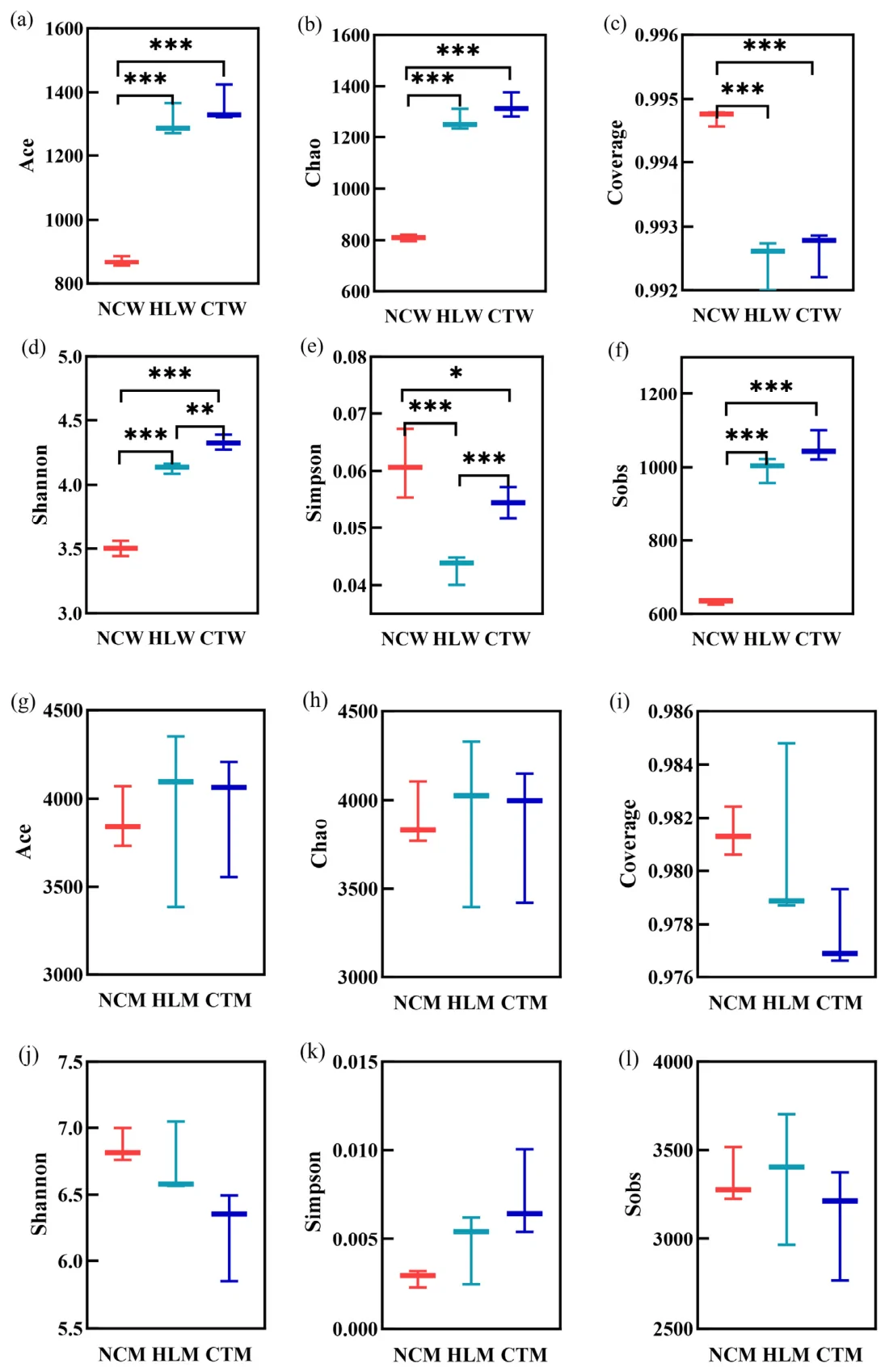

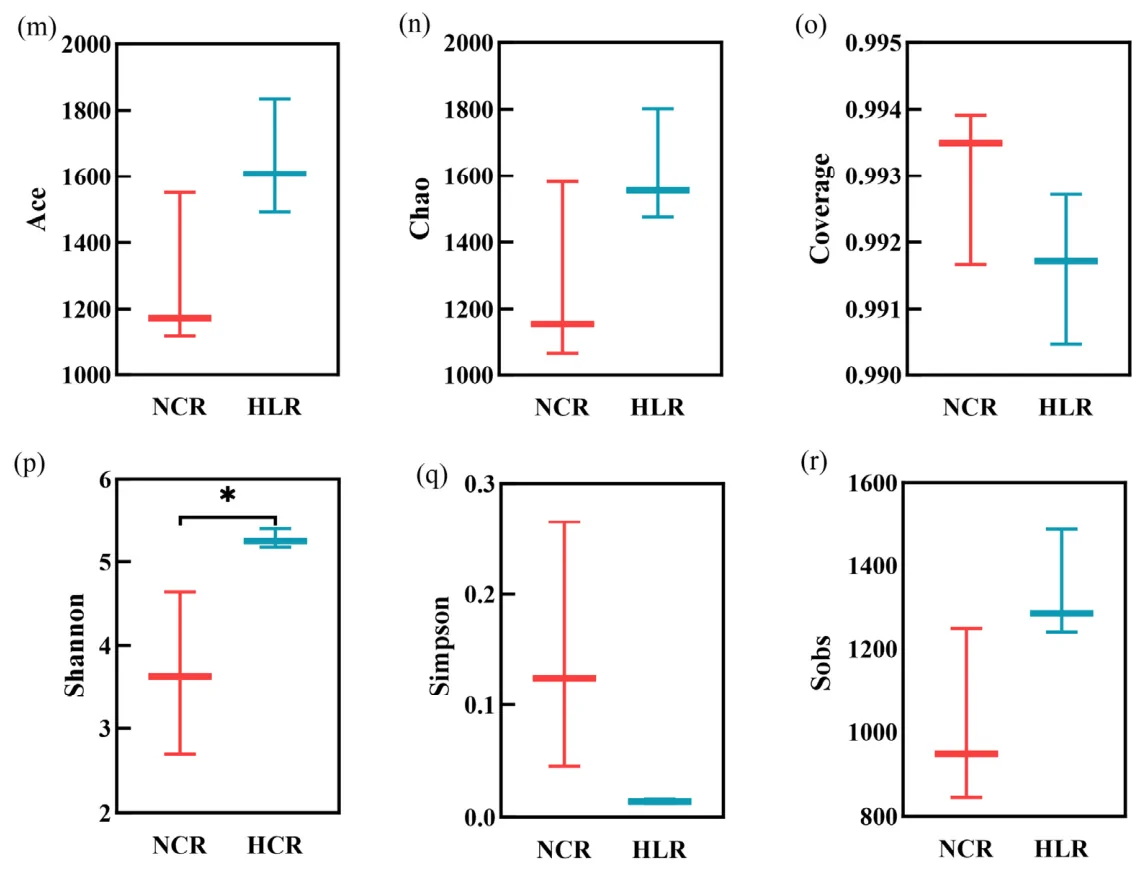
Alpha diversity indices of microbial communities under different treatments. (**a**–**f**) ACE, Chao, Coverage, Shannon, Simpson and Sobs indices of planktonic microbial communities; (**g**–**l**) ACE, Chao, Coverage, Shannon, Simpson and Sobs indices of sediment microbial communities; (**m**–**r**) ACE, Chao, Coverage, Shannon, Simpson and Sobs indices of rhizosphere microbial communities. * (0.01 ≤ *p* < 0.05), ** (0.001 ≤ *p* < 0.01), *** (*p* < 0.001).

Microbial diversity analysis of 42 samples yielded a total of 2,553,488 optimized sequences and 1,061,431,932 bases, with an average sequence length of 415 bp, resulting in 12,348 OTUs classified into 60 phyla, 188 classes, 459 orders, 750 families, 1459 genera, and 3312 species. As shown in Figure 3, NCW, HLW, and CTW represented planktonic bacteria in the *Nepeta cataria* group, *Lactuca sativa* group, and no-plant group, respectively; NCM, HLM, and CTM represented sediment bacteria in the *Nepeta cataria* group, *Lactuca sativa* group, and no-plant group, respectively; NCR and HLR represented rhizosphere bacteria in the *Nepeta cataria* group and *Lactuca sativa* group, respectively. The coverage of all samples was higher than 0.99 (Figure 3c,i,o). The alpha diversity of the planktonic bacterial communities in the three groups was shown in Figure 3. The order of the ACE, Chao, and Sobs indices for the planktonic bacteria in the *Nepeta cataria* group (NCW), the *Lactuca sativa* group (HLW), and the no-plant group (CTW) was consistently: NCW < HLW < CTW (Figure 3a,b,f). Furthermore, the ACE, Chao, and Sobs indices of NCW were significantly lower than those of HLW and CTW (*p* < 0.001), indicating that HLW and CTW possessed richer bacterial communities. The Shannon and Simpson indices differed significantly among the three groups (*p* < 0.05), with the highest Shannon index in the CTW group and the lowest in the NCW group (Figure 3d,e), indicating higher community stability in the CTW group. Except for the Shannon indices of rhizosphere bacteria in the *Nepeta cataria* group (NCR) and the *Lactuca sativa* group (HLR) (Figure 3p), there were no significant differences in the alpha diversity indices of sediment and rhizosphere bacterial communities across groups.

**Figure 4:**
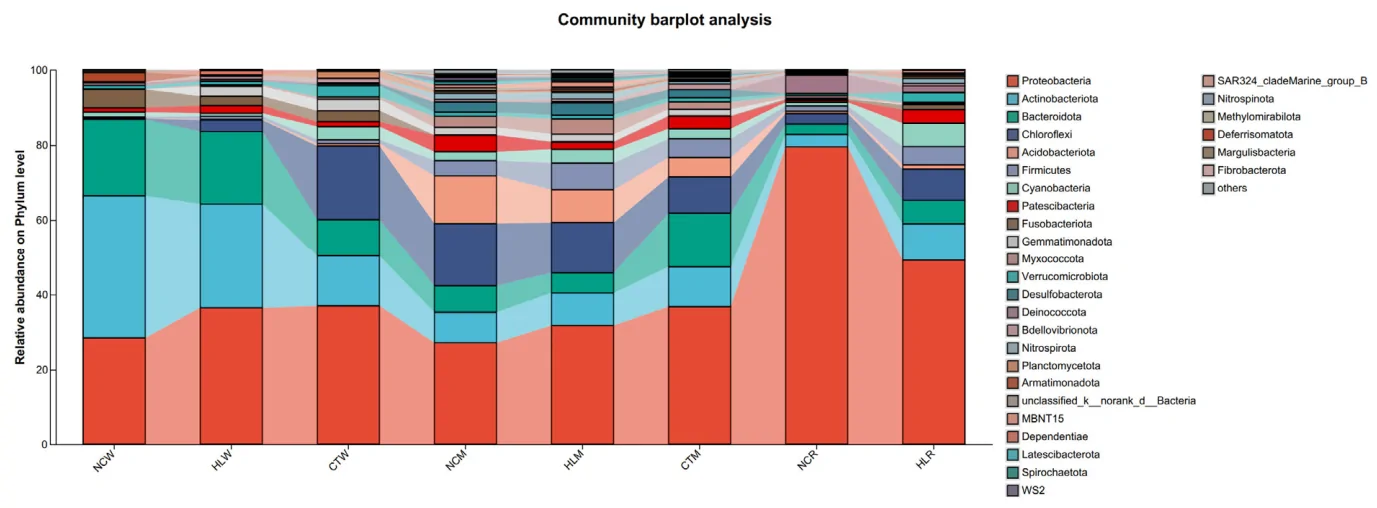
Microbial composition at the phylum level.

The top 30 phyla in relative abundance were similar across all groups, but differences were observed in their relative abundances (Figure 4). The NCR *Proteobacteria* phylum had the highest relative abundance, reaching 79.50%, followed by the HLR at 49.12%. The phyla *Actinobacteria* and *Bacteroidota* were dominant in CTW, HLR, and NCW, and both showed a gradual increase, rising from 13.36% to 38.02% and from 9.81% to 20.38%, respectively. The *Acidobacteriota* phylum was the dominant phylum in CTM, HLM, and NCM, showing a gradual increase from 5.16% to 12.67%. In addition, the *Chloroflexi* phylum was a common dominant phylum in NCM, HLM, CTM, HLR, and CTW, with relative abundances of 16.74%, 13.56%, 9.67%, 8.25%, and 19.58%, respectively. It was worth noting that the phylum *Nitrospirota* was detected in NCM, HLM, and HLR, with relative abundances of 1.61%, 1.74%, and 1.37%, respectively.

**Figure 5:**
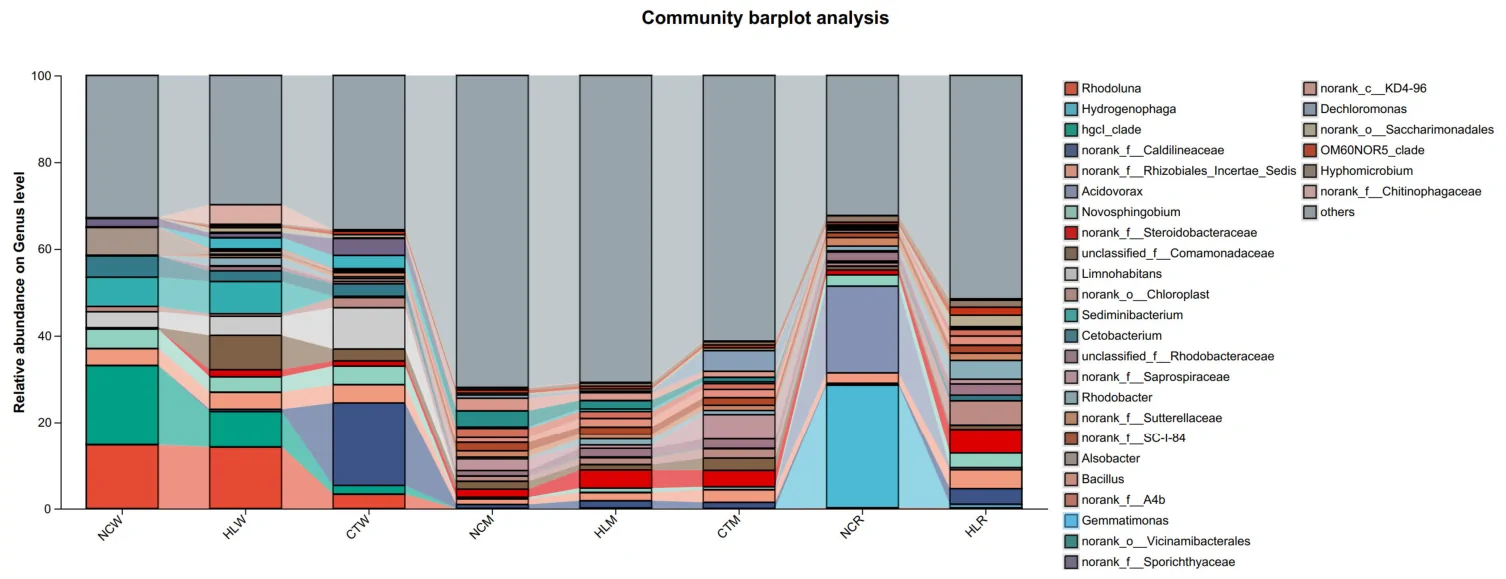
Genus-level microbial composition.

The composition of microbial groups at the genus level differed among the groups (Figure 5). hgcI_clade, *Rhodoluna*, and *Sediminibacterium* were the dominant genera in NCW and HLW. The relative abundances of hgcI_clade in NCW and HLW were 18.43% and 8.33%, respectively; those of *Rhodoluna* were 14.65% and 14.15%, respectively; and those of *Sediminibacterium* were 6.74% and 7.45%, respectively. The relative abundances of hgcI_clade, *Rhodoluna*, and *Sediminibacterium* all decreased in CTW, dropping to 1.98%, 3.33%, and 0.29%, respectively. *Alsobacter* was the dominant genus in NCW, with a relative abundance of 6.38%; its relative abundance in the other groups was below 1%. unclassified_f_*Comamonadaceae* and norank_f_*Chitinophagaceae* were the dominant genera in HLW, with relative abundances of 7.92% and 4.53%, respectively, higher than in other groups. norank_f_*Caldilineaceae* was the dominant genus in CTW, with a relative abundance of 19.18%; its relative abundance in both HLW and NCW was less than 1%. The relative abundance of *Limnohabitans* showed a gradual increase across NCW, HLW, and CTW, rising from 3.67% to 9.53%. The relative abundances of *Gemmatimonas* in HLW and CTW were 3.03% and 2.55%, respectively, higher than in NCW. *Hydrogenophaga* and *Acidovorax* were the dominant genera in NCR, with relative abundances of 28.42% and 20.00%, respectively; their relative abundances in HLR and CT were both below 1%. norank_o_*Chloroplast* was the dominant genus in HLR, with a relative abundance of 5.61%, higher than in other groups. *Dechloromonas* and norank_f_*Saprospiraceae* were the dominant genera in CTM, with relative abundances of 4.78% and 5.72%, respectively, higher than in other groups.

**Figure 6:**
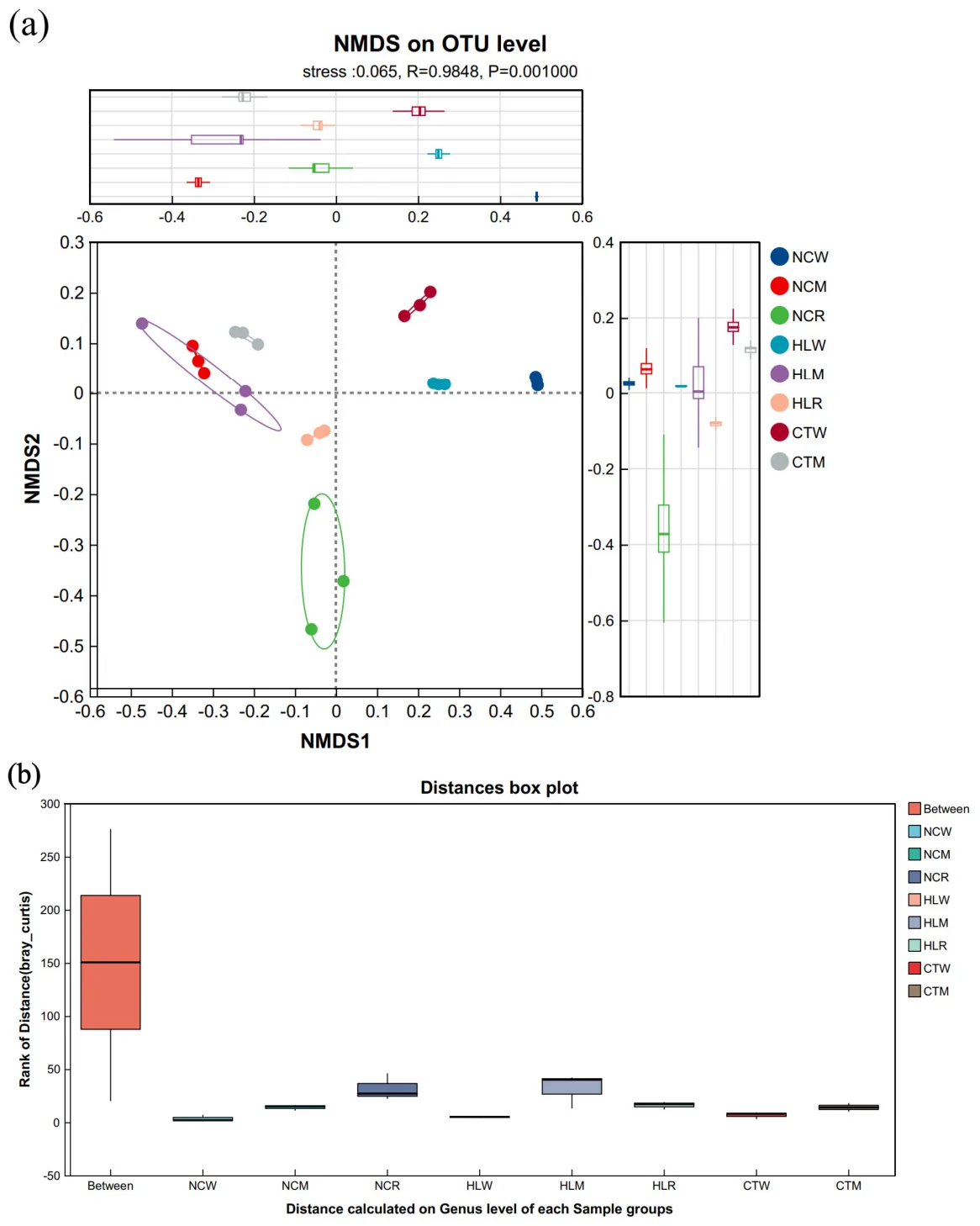
NMDS Analysis of Microbial Communities. (**a**) NMDS on OUT level; (**b**) Distances box plot.

To investigate the distribution characteristics and patterns of change in microbial communities between the experimental and control groups, NMDS and ANOSIM analyses were performed based on Bray–Curtis distance. Figure 6 showed that samples from the sediment, rhizosphere, and water of each group clustered together and exhibited high similarity, indicating that substrate type was the primary driver influencing the structure of microbial communities in aquaculture water (Figure 6a). The ANOSIM analysis results indicated that the between-group differences in microbial communities among the three groups were greater than the within-group differences (*R*(Statistic) = 0.9778, *p* < 0.05) (Figure 6b). Additionally, samples from sediment, rhizosphere, and water in each group could be distinguished from one another, suggesting that grouping also influenced microbial community structure. The results of the Adonis analysis (*R*^2^ = 0.9224, *p* < 0.05) validated the above conclusions (Table 1 and Table 2).

### 3.3. Correlation Analysis Between Physicochemical Properties and Microbial Community Structure

**Figure 7:**
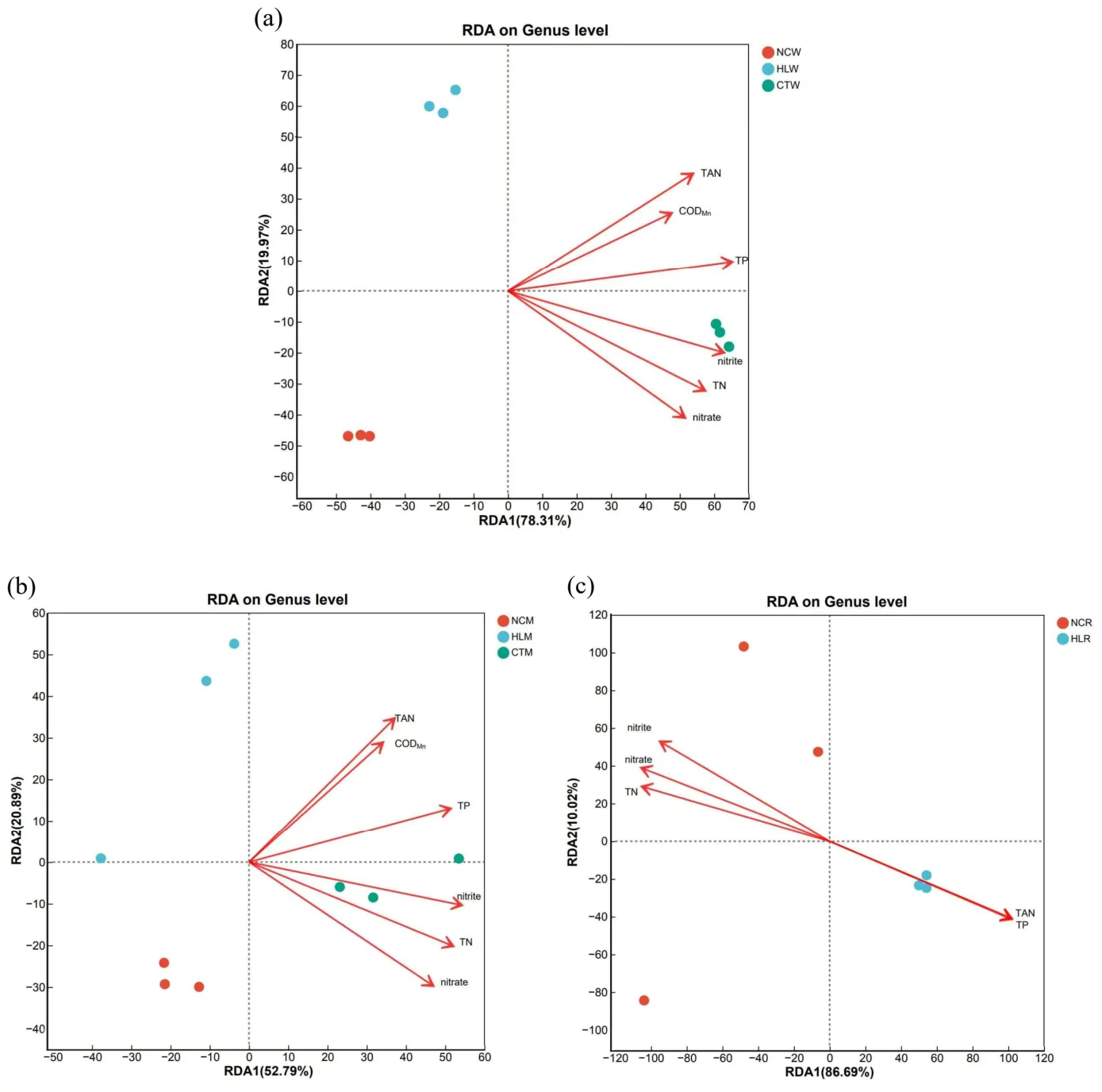
RDA Analysis of Microbial Communities. (**a**) RDA for planktonic microorganisms; (**b**) RDA for sedimentary microorganisms; (**c**) RDA for rhizosphere microorganisms.

To further analyze the relationship between microbial structure and environmental factors, a Reduced Dimension Analysis (RDA) based on Pearson distance was conducted (Figure 7). The results showed that for planktonic microorganisms, the explanatory proportions of the first and second axes were 78.31% and 19.97% (Figure 7a). Samples from the NC group were positioned opposite to the vectors of TAN, COD_Mn_, and TP, indicating a negative association with these variables. HL samples showed negative associations with nitrite, nitrate, and TN, whereas CT samples were positively associated with nitrite, nitrate, and TN. The envfit test for environmental factors indicated that planktonic microbial community structure was significantly shaped by TAN (r^2^ = 0.99808, *p* = 0.001), nitrite (r^2^ = 0.9967, *p* = 0.003), nitrate (r^2^ = 0.99151, *p* = 0.003), TP (r^2^ = 0.99702, *p* = 0.004), and TN (r^2^ = 0.9918, *p* = 0.005) (*p* < 0.05).

For sedimentary microorganisms, the explanatory power of the first and second axes was 52.79% and 20.89%, respectively (Figure 7b). The influence of environmental factors on the microbial community composition in the sediment was similar to that observed in the planktonic bacterial community. The envfit test for environmental factors indicated that sedimentary microbial community structure was significantly shaped by TAN (r^2^ = 0.68624, *p* = 0.031), nitrite (r^2^ = 0.89284, *p* = 0.015), nitrate (r^2^ = 0.9334, *p* = 0.002), TP (r^2^ = 0.77766, *p* = 0.023), and TN (r^2^ = 0.93016, *p* = 0.001) (*p* < 0.05).

For rhizosphere microorganisms, the explanatory power of the first and second axes was 86.69% and 10.02%, respectively (Figure 7c). NC rhizosphere samples exhibited negative associations with TAN and TP, while HL rhizosphere samples were negatively associated with nitrite, nitrate, and TN. The envfit test for environmental factors indicated that rhizosphere microbial community structure was significantly shaped by nitrite (r^2^ = 0.4863, *p* = 0.03889), nitrate (r^2^ = 0.95088, *p* = 0.0111), and TN (r^2^ = 0.89384, *p* = 0.0444) (*p* < 0.05).

**Figure 8:**
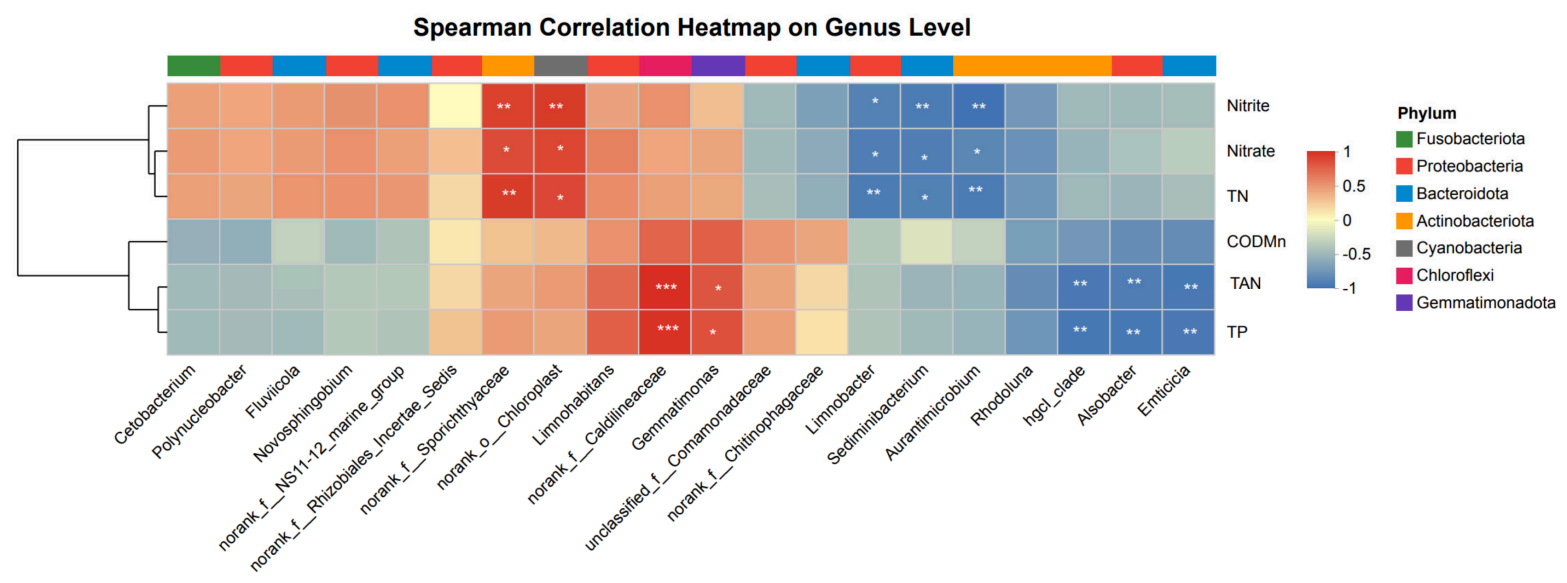
Spearman correlation heatmap of planktonic microorganisms and environmental factors. Significance of correlations was determined based on Benjamini–Hochberg FDR-adjusted *p*-values. * (0.01 ≤ *p* < 0.05), ** (0.001 ≤ *p* < 0.01), *** (*p* < 0.001).

The Spearman correlation heatmap between dominant genera and environmental factors was shown in Figure 8. It was observed that, among planktonic microorganisms, *Limnobacter*, *Sediminibacterium*, and *Aurantimicrobium* exhibited highly significant negative correlations with nitrite, nitrate, and TN. *Alsobacter*, hgcI_clade, and *Emticicia* showed extremely significant negative correlations with TAN and TP. *Sediminibacterium*, *Limnobacter*, and *Aurantimicrobium* showed significant negative correlations with nitrite, nitrate, and TN. In contrast, norank_f_*Sporichthyaceae* and norank_o_*Chloroplast* showed extremely significant positive correlations with nitrite, nitrate, and TN. Norank_f_*Caldilineaceae* and *Gemmatimonas* showed significant positive correlations with TAN and TP.

**Figure 9:**
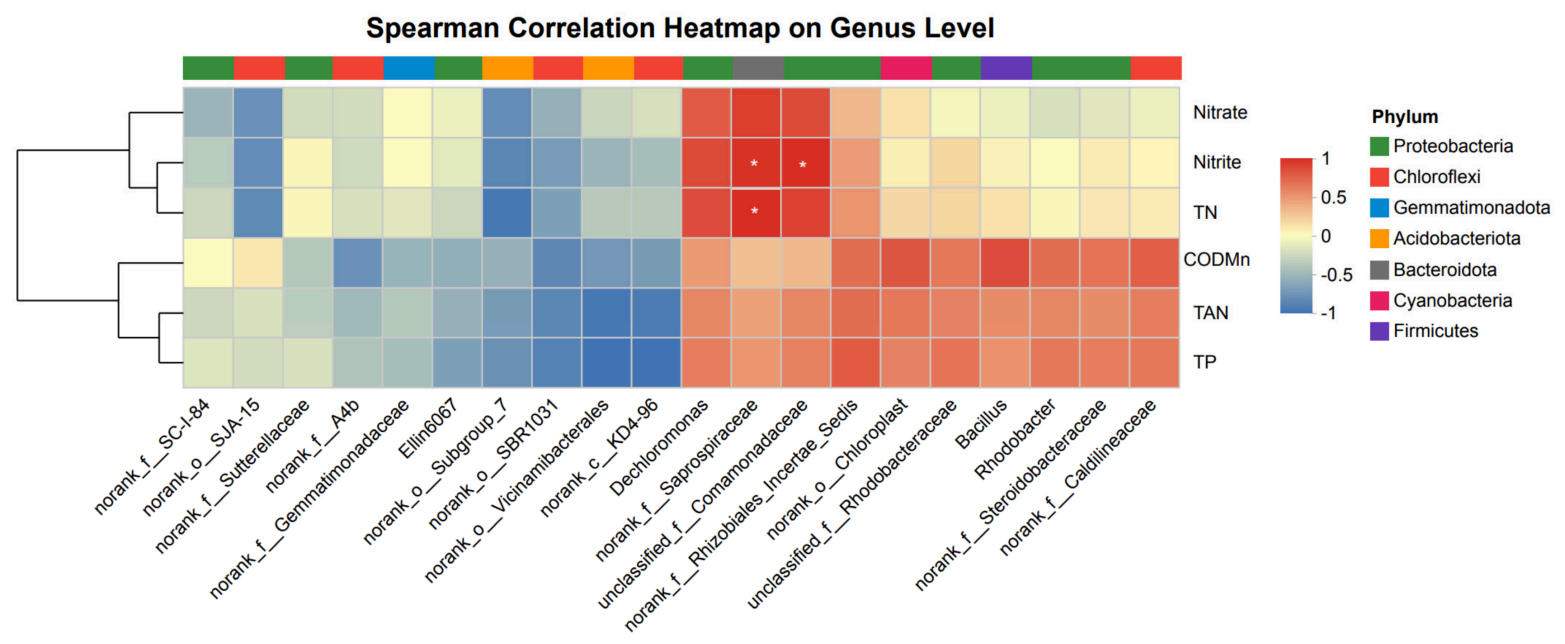
Spearman correlation heatmap of sedimentary microorganisms and environmental factors. Significance of correlations was determined based on Benjamini–Hochberg FDR-adjusted *p*-values. * (0.01 ≤ *p* < 0.05).

Among bottom sediment microorganisms (Figure 9), norank_f_*Saprospiraceae* and unclassified_f_*Comamonadaceae* were significantly positively correlated with nitrite and TN; unclassified_f_Comamonadaceae was significantly positively correlated with nitrite.

**Figure 10:**
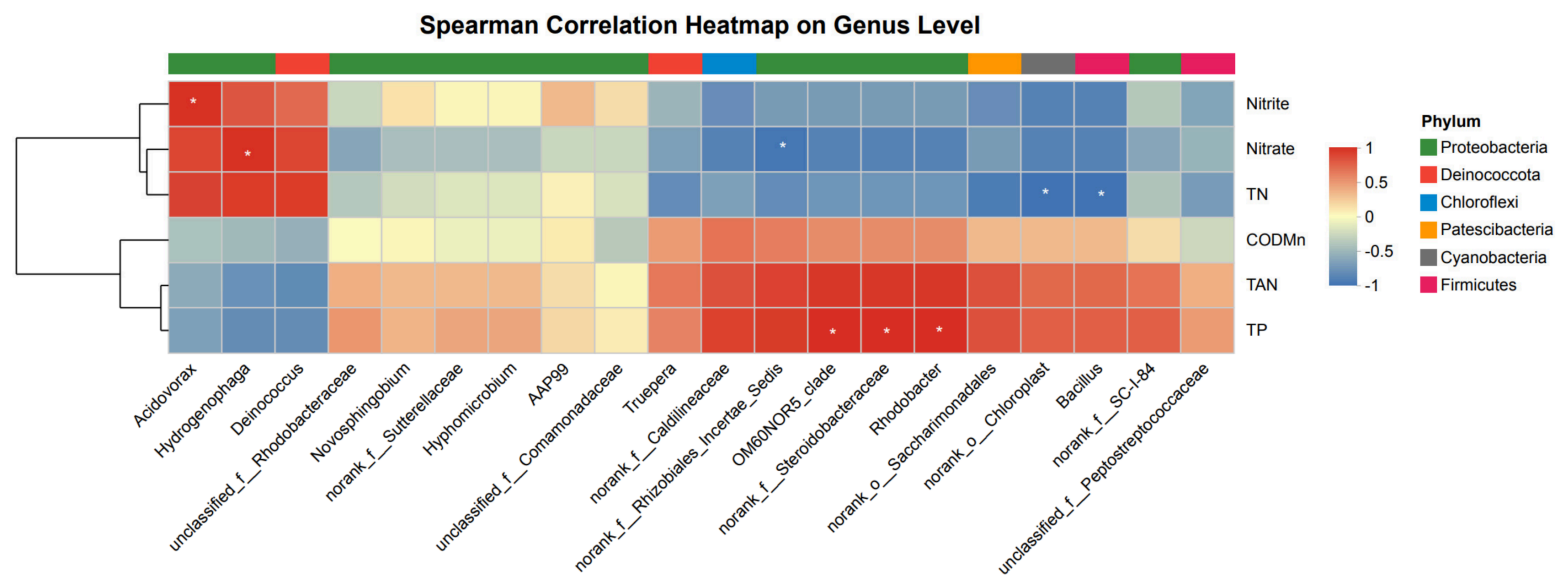
Spearman correlation heatmap of rhizosphere microorganisms and environmental factors. Significance of correlations was determined based on Benjamini–Hochberg FDR-adjusted *p*-values. * (0.01 ≤ *p* < 0.05).

Among rhizosphere microorganisms (Figure 10), *Acidovorax* was significantly positively correlated with nitrite. Norank_o_*Chloroplast* and *Bacillus* were significantly negatively correlated with TN. *Hydrogenophaga* was significantly positively correlated with nitrate, while g__norank_f__Rhizobiales_Incertae_Sedis was significantly negatively correlated with nitrate. OM60NOR5_clade, norank_f_*Steroidobacteraceae*, and *Rhodobacter* were significantly negatively correlated with TP.

## 4. Discussion

### 4.1. Effects of Nepeta cataria and Lactuca sativa on Nitrogen and Phosphorus Parameters in Aquaculture Water

The results of this study indicated that, compared to the group without planted vegetation, both *Nepeta cataria* and *Lactuca sativa* significantly reduced the levels of nitrogen and phosphorus environmental factors in the aquaculture water. Specifically, the *Nepeta cataria* group had the lowest levels of TAN and TP, while the *Lactuca sativa* group demonstrated equally significant control effects on nitrite, nitrate, and TP. This confirmed that both plant species can effectively improve the nitrogen and phosphorus nutrient status of aquaculture water. These findings were consistent with the mechanism by which plants in aquaponics systems achieve water purification through direct absorption, root accumulation, and enhanced microbial transformation [9,12].

**Figure 11:**
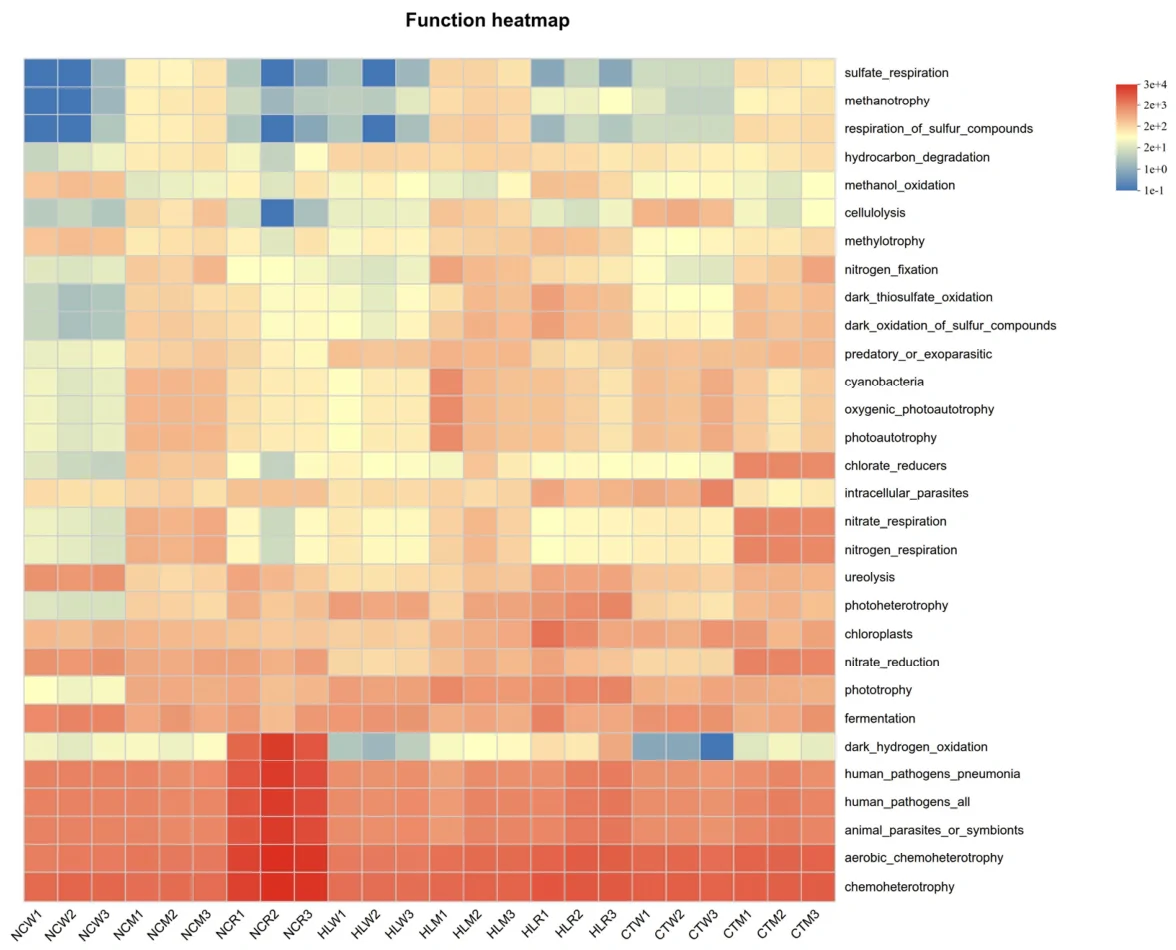
Functional Prediction Heatmap of Microbial Communities. Note: Functional potentials were predicted from 16S rRNA gene data using FAPROTAX, not directly quantified.

At the microbial level, the phylum *Proteobacteria* was significantly enriched in the rhizosphere of both *Nepeta cataria* and *Lactuca sativa*, particularly in the *Nepeta cataria* rhizosphere, where it reached as high as 79.50%, suggesting that the plant rhizosphere may provide a critical microhabitat for nitrogen-transforming microbial communities. As a core group in nitrogen metabolism, the *Proteobacteria* are extensively involved in ammonia oxidation, nitrate reduction, and denitrification processes [11], and their increased abundance is closely associated with enhanced denitrification capacity in both the *Nepeta cataria* and *Lactuca sativa* groups. This study found that *Alsobacter* was highly significantly negatively correlated with TAN and TP, and significantly negatively correlated with COD_Mn_. *Alsobacter* is a genus of Gram-negative aerobic bacilli within the *Proteobacteria*. In recent years, it has been frequently detected in estuarine sediments and aquaculture waters. Its core ecological functions are concentrated in denitrification, sulfate reduction, and organic matter degradation [19], playing a crucial role in nitrogen–sulfur coupling cycles and pollution remediation in aquatic environments. Concurrently, the abundances of the phyla *Actinobacteriota* and *Bacteroidota* in the water samples exhibited a trend of CTW < HLW < NCW, further reinforcing the increasing trend in nitrogen cycling efficiency observed in the *Nepeta cataria* and *Lactuca sativa* groups (Figure 11). These two microbial communities can promote organic nitrogen mineralization, nitrate reduction, and the degradation of complex organic matter through synergistic metabolism [19,20]. This study found that the hgcI_clade within the *Actinobacteriota* phylum was a key core bacterium in the *Nepeta cataria* group and showed significant negative correlations with TAN and TP, nitrate, and TP, which is consistent with the findings of Wang, B. et al. [21]. The hgcI_clade possesses genes related to denitrification and nitrogen fixation and serves as a bacterial indicator taxon driving differences in community responses along the wastewater concentration gradient [22].

The abundance of the *Acidobacteriota* phylum in sediments gradually increased with plant colonization. This group encodes functional genes involved in nitrate reduction and nitrite reduction, participating in denitrification and organic nitrogen transformation in anoxic microenvironments [23], which helps reduce nitrate levels. The aerobic-anaerobic environment present in plant root zones is conducive to the growth of *Acidobacteriota*.

In aquaponics systems, ammonia-oxidizing microorganisms (AOMs) primarily grow and reproduce in and around plant roots and are easily regulated by plants [24]. The rhizosphere is a complex zone of plant–microbe interactions [25], whose formation is influenced by root exudates; these exudates act as signaling molecules that regulate microbial composition and biogeochemical processes [26]. Plant-mediated regulation selectively influences ammonia-oxidizing microorganisms through various pathways, such as the release of biological nitrification inhibitors (BNIs) and the alteration of key niche determinants like pH and available carbon [27,28,29]. A growing body of empirical evidence indicates that these processes selectively promote the growth of complete ammonia-oxidizing bacteria (Comammox). This is consistent with the findings of this study, in which the phylum *Nitrospirota* was detected in the sediments of the *Nepeta cataria* group, the *Lactuca sativa* group, and the *Lactuca sativa* rhizosphere. As a typical nitrifying microbial community, *Nitrospirota* drives nitrite oxidation and the entire nitrification process [9], making nitrogen transformation more stable and complete, thereby reducing the accumulation of toxic intermediate products such as nitrite. This is confirmed by the significantly lower NITRITE content in the NC and HL groups compared to the CT group.

In addition to modulating microbial community composition, plant root exudates contribute additional organic substances in the treatment groups. While root exudates enhanced organic matter mineralization via microbial regulation, they also added organic matter to the system, which may account for the statistically non-significant difference in COD_Mn_ between treatment and control groups.

### 4.2. Regulation of Microbial Community Structure in Aquaculture Water by Nepeta cataria and Lactuca sativa

**Figure 12:**
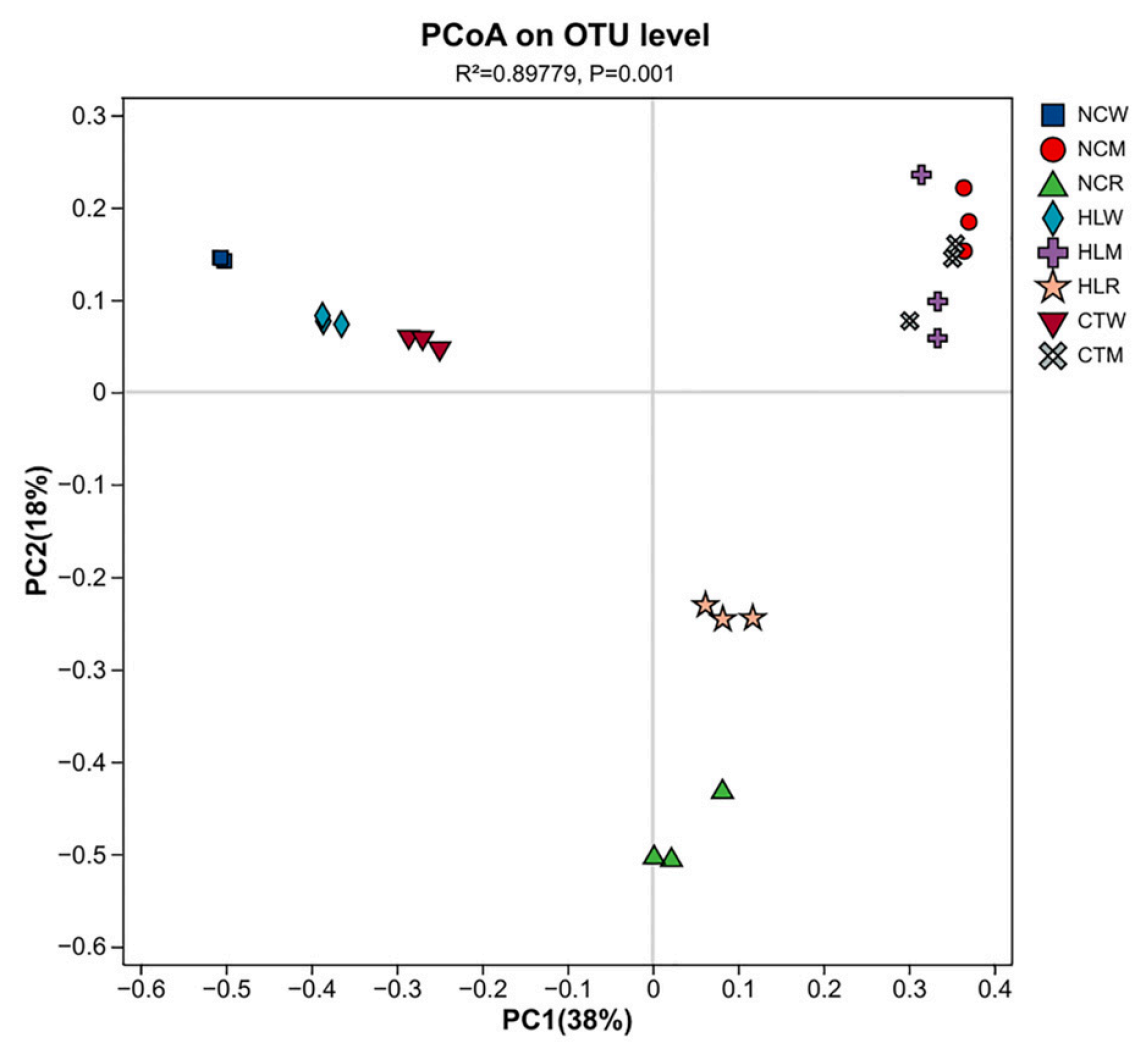
Principal coordinate analysis (PCoA) of microbial communities at the OTU level. Notes: NCW, NCM and NCR represent planktonic, sediment and rhizosphere microorganisms in the *Nepeta cataria* group, respectively. HLW, HLM and HLR represent planktonic, sediment and rhizosphere microorganisms in the lettuce group, respectively. CTW and CTM represent planktonic and sediment microorganisms in the control group.

Rhizosphere exudates shape microbial community structure [30]. The alpha diversity results of this study showed that the diversity of the rhizosphere microbial community in the *Nepeta cataria* group was significantly lower than that in the *Lactuca sativa* group, and the abundance of the planktonic bacterial community in the *Nepeta cataria* group was extremely significantly lower than that in the *Lactuca sativa* group and the non-planted group. Diversity exhibited a trend of NC < HL < CT, while there was no significant difference in the abundance and diversity of the sediment microbial community. PCoA results revealed clear spatial separation of microbial communities across different treatment groups (Figure 12). PERMANOVA confirmed that the treatments significantly altered microbial community structure (R^2^ = 0.89779, *p* = 0.001), indicating that the experimental treatments served as the primary driver of community differentiation (Figure 12). PC1 and PC2 together explained 56% of the total community variation, and samples were mainly differentiated along the PC1 axis, suggesting that the environmental gradient corresponding to PC1 was the core factor shaping microbial community assembly. Tight clustering of replicates within each group reflected high stability and reproducibility of microbial communities under the same treatment. Such differences in community structure imply that distinct treatment conditions select microorganisms by modifying the physicochemical properties of habitats, thereby regulating community composition.

The significantly reduced richness and diversity of rhizosphere and planktonic microorganisms in the *Nepeta cataria* group indicated that *Nepeta cataria* exerts a strong selective shaping effect on rhizosphere and planktonic bacterial communities. *Lactuca sativa* root exudates consist of amino acids, sugars, and organic acids [31], whereas the main components of *Nepeta cataria* are monoterpenes and sesquiterpenes [32]. These metabolites may serve as both nutrient sources and signaling molecules, creating favorable niches for specific microbial groups [33] and regulating the growth of bacteria and fungi [34]; however, they may also reduce the stability of microbial communities [35], which is consistent with the findings of this study.

NMDS, ANOSIM, and Adonis analyses further confirmed that different groups and different substrate samples were significantly separated, with highly significant differences between groups, indicating that plants can reshape the microbial communities in aquaculture water bodies at the structural level. Studies have shown that in aquaponics systems, there are complex interactions between rhizosphere microorganisms and aquatic microorganisms. The two are regulated through root exudates [36,37,38], diffusion and migration [39], synergistic effects [40], and community feedback loops to jointly drive the system’s nitrogen cycle and nutrient transformation. Plants selectively recruit specific microbial groups through root exudates (such as organic acids and secondary metabolites), forming root-specific microbial communities, such as the enrichment of *Proteobacteria* and *Actinobacteria*. These microorganisms directly promote nutrient uptake through nitrogen fixation and phosphorus solubilization genes (e.g., *nifH*, *phoD*) [41], thereby altering aquatic environmental factors. This study found that both *Nepeta cataria* and *Lactuca sativa* significantly increased the relative abundance of functional groups such as the *Actinobacteria*, *Bacteroidetes*, and *Acidobacteria* phyla, exhibiting a trend of NC > HL > CT. Multiple studies have demonstrated that Proteobacteria and Bacteroidetes are common taxa in plant roots within aquaponic systems [42,43,44], which is consistent with our findings. At the genus level, hgcI_clade, *Rhodoluna*, and *Sediminibacterium* emerged as signature dominant genera in the floating water of the *Nepeta cataria* and *Lactuca sativa* groups, whereas their abundance decreased significantly in the control group. As a typical oligotrophic, highly efficient carbon- and nitrogen-utilizing group in freshwater environments, hgcI_clade was significantly enriched in the plant groups, indicating that plants improved the nutrient structure of the water through root exudates and nutrient uptake, thereby promoting the proliferation of highly metabolically active bacterial communities. *Rhodoluna*, also a typical planktonic bacterium within the Actinobacteria phylum, possesses characteristics such as a reduced genome and efficient substrate utilization [45,46,47], and together with hgcI_clade, constitutes the core functional bacterial community of the plant group.

Most *Bacteroidota* are capable of gliding motility, a trait mediated by their unique type IX secretion system (T9SS) and the *gld/spr* gene family [48], enabling them to efficiently adhere to and colonize suspended particles, sediments, and cell surfaces, forming structurally stable biofilm communities [49]. The well-developed root systems of hydroponic plants provide a vast surface area for *Bacteroidota* to attach to. At the microinterface, *Bacteroidota* secrete carbohydrate-active enzymes (CAZymes) [50] and extracellular polymers (EPS) [51], driving the degradation of particulate organic matter (POM) and the recycling of nutrients. They serve as a key functional group linking primary production to the heterotrophic food web and synergistically driving nitrogen and phosphorus removal [52].

Furthermore, the floating water column in the *Nepeta cataria* group was uniquely enriched with *Alsobacter*, while the *Lactuca sativa* group was enriched with the *Monilaceae* and *Chondrosporaceae* families; the control group was dominated by the *Caldilineaceae* family. Such taxonomic discrepancy demonstrates that host-plant filtering effects arising from divergent root-exudate profiles, root oxygen-releasing potentials, and nutrient-acquisition strategies can select and recruit distinct functional bacterial consortia. Root-exudate chemical constituents deliver variable carbon substrates for heterotrophic microbes, while root oxygen leakage modulates rhizosphere and surrounding water-column redox microhabitats, which further determines the relative competitive advantages of aerobic, facultative-anaerobic, and anaerobic functional populations. Coupled with plant-driven nutrient depletion in the surrounding water, these multi-faceted plant traits jointly drive the taxonomic divergence of planktonic microbial communities. Consequently, distinct nitrogen- and phosphorus-removal pathways are shaped in different plant-based systems. Specifically, the enrichment of *Alsobacter* in the NC water column may contribute to its superior TAN and TP removal performance, while the enrichment of *Monilaceae* and *Chondrosporaceae*-related taxa in the HL group is likely linked to its strong capability for nitrate and nitrite transformation. Nevertheless, since these observations are derived solely from terminal-time-point sampling, the dynamic causal relationships between plant physiological traits and the succession of these specialized bacterial groups cannot be fully elucidated in the present study. Further controlled experiments are needed to disentangle the individual contributions of root exudates, oxygen release, and nutrient uptake to community assembly.

### 4.3. Coupling Relationships Between Microbial Communities and Nitrogen and Phosphorus Environmental Factors

RDA and Spearman correlation analyses based on the terminal sampling dataset indicated that nitrogen and phosphorus environmental factors were key drivers of microbial community structure variation. The planktonic bacteria community in the *Nepeta cataria* group showed a significant negative correlation with TAN, TP, and COD_Mn_, while the *Lactuca sativa* group exhibited a negative correlation with nitrite, nitrate, and TN (Figure 7a). In contrast, the control group showed a positive correlation with these parameters, demonstrating that the observed differentiation in microbial community structure at the end of cultivation corresponded to differences in the nitrogen and phosphorus transformation capacity among experimental systems.

In the surface water column, hgcI_clade, *Alsobacter*, and *Emticicia* show a highly significant negative correlation with TAN and TP, indicating that these taxa may play a key role in the reduction in ammonia nitrogen and total phosphorus at the sampling endpoint. The hgcI_clade possesses a strong ability to absorb carbohydrates and nitrogen-rich compounds (Trevor W Ghylin et al., 2014 [22]). *Sediminibacterium*, *Limnobacter*, and others showed significant negative correlations with nitrite, nitrate, and TN, suggesting their potential involvement in the reduction of nitrate and nitrite nitrogen. Collectively, these observations support a plausible hypothesis that plants may achieve water purification by suppressing eutrophication-indicator bacterial groups and enriching nitrogen and phosphorus-transforming functional bacterial groups, though we cannot quantify the relative contributions of plant uptake versus microbial transformation in the present study.

Sediment and rhizosphere microorganisms also exhibited significant responses to environmental factors measured at the final sampling time point. Among rhizosphere microorganisms, genera such as *Hydrogenophaga* and *Acidovorax* were highly abundant in the *Nepeta cataria* group and showed significant correlations with nitrogen forms, indicating that the *Nepeta cataria* rhizosphere may facilitate the enrichment of denitrifying and nitrate-reducing bacterial communities, which could enhance the system’s denitrification potential. For the HL group with the lowest TN concentration, two signature genera, unclassified_f_Comamonadaceae and norank_f_Chitinophagaceae, were significantly enriched in water samples. Members within Comamonadaceae possess complete sets of nitrate-reduction and denitrification genes [53], which may facilitate the transformation of accumulated nitrate and nitrite. This provides a potential explanation for the superior TN removal capacity of HL. Distinct nitrogen–phosphorus removal pathways exist between the two plant groups: HL may mainly achieve TN reduction via nitrate–nitrite-transforming functional bacteria, while NC may gain advantages in TAN and TP elimination by combining high-rate plant absorption and rhizosphere denitrifier enrichment. Proteobacteria were enriched in the NC group at the final sampling time point. Within this phylum, *Hydrogenophaga* and *Acidovorax* were dominant, with relative abundances of 28.42% and 20.00%, respectively. *Hydrogenophaga* mediates hydrogenotrophic denitrification to convert nitrate and TAN into gaseous nitrogen compounds, and *Acidovorax* harbors heterotrophic nitrate reduction pathways [54]. Massive enrichment of these two genera may potentially enhance rhizosphere nitrogen transformation.

Overall, the coupling relationship between microbial communities and environmental factors observed at the end of the culture period supports the hypothesis that *Nepeta cataria* and *Lactuca sativa* may alter water nutrient levels to selectively recruit functional microorganisms, forming a “plant–microbe” synergistic purification mechanism. We emphasize that this represents a hypothetical model rather than a fully proven mechanistic finding, because our current dataset cannot quantitatively partition the relative contributions of plant nutrient uptake and microbial transformation. This hypothesized interaction may underpin the stability and efficiency of aquaponics systems. It should be noted that water and microbial samples were collected only once on the final day (July 23) in this study. Therefore, the dynamic relationships among variations in nitrogen and phosphorus concentrations, microbial community succession, and plant growth cycles cannot be reflected. Future studies should supplement dynamic monitoring data collected during the middle of the culture period to further clarify the temporal patterns of plant–microbe synergistic purification.

## 5. Conclusions

Both *Nepeta cataria* and *Lactuca sativa* effectively mitigated nitrogen and phosphorus pollution in the aquaponic system and exhibited complementary purification effects. *Nepeta cataria* demonstrated superior removal performance for TAN and TP, whereas *Lactuca sativa* showed a greater capacity to reduce nitrite-N and nitrate-N. Substrate type (water column, sediment, and rhizosphere) was the primary determinant of microbial community structure. *Nepeta cataria* exerted stronger selective pressure on planktonic bacterial communities, resulting in significantly lower richness and diversity than in the *Lactuca sativa* and no-plant control groups. Both plant species enriched key functional microbial taxa, including Proteobacteria, Actinobacteriota, Bacteroidota, Acidobacteriota, and Nitrospirota. The enrichment of these taxa may facilitate nitrification–denitrification coupling and nutrient transformation. Our observations support a plausible hypothesis of a synergistic “plant uptake–microbial transformation” purification model, rather than confirming this mechanism.

However, as a short-term study based on 16S rRNA gene amplicon sequencing, the relative contributions of plant uptake and microbial transformation were not quantified. Collectively, our findings provide theoretical support for plant species selection and ecological regulation in floating-bed aquaponics. Further studies incorporating metagenomic sequencing and multifactor controlled trials are needed to elucidate the underlying mechanisms.

## Figures and Tables

**Table 1:** ANOSIM A.

Method	Statistic	*p*-Value	Permutation_Number
ANOSIM	0.9778	0.001	999

**Table 2:** Adonis Analysis Results.

	Df	Sums of Sqs	Mean Sqs	F.Model	*R* ^2^	Pr (>F)
Group (factor$group3)	7	4.9356	0.7051	27.17916	0.9224	0.001
Residuals	16	0.4151	0.0259		0.0776	
Total	23	5.3506			1	

## Data Availability

The original contributions presented in this study are included in the article. Further inquiries can be directed to the corresponding author.

## References

[1] Fishery and Fishery Administration Bureau, Ministry of Agriculture and Rural Affairs (2026). 2025 National Fishery Economic Statistical Bulletin.

[2] Ministry of Agriculture and Rural Affairs, Ministry of Ecology and Environment (2025). China Fishery Ecological Environment Status Bulletin (2024).

[3] Conley D.J., Paerl H.W., Howarth R.W., Boesch D.F., Seitzinger S.P., Havens K.E., Lancelot C., Likens G.E. (2009). Controlling eutrophication: Nitrogen and phosphorus. Science.

[4] Smith V.H., Schindler D.W. (2009). Eutrophication science: Where do we go from here?. Trends Ecol. Evol..

[5] Xiao K., Hao J.W., Wang Y., Qu B.C., Fu S.Z., Liu Y. (2021). Effects of microfiltration in recirculating aquaculture system on bacterial community structure in water. Fish. Mod..

[6] Gonçalves A.A., Gagnon G.A. (2011). Ozone Application in Recirculating Aquaculture System: An Overview. Ozone Sci. Eng..

[7] Schröder J.P., Croot P.L., von Dewitz B., Waller U., Hanel R. (2011). Potential and Limitations of Ozone for the Removal of Ammonia, Nitrite, and Yellow Substances in Marine Recirculating Aquaculture Systems. Aquac. Eng..

[8] Baganz G.F.M., Junge R., Portella M.C., Goddek S., Keesman K.J., Baganz D., Staaks G., Shaw C., Lohrberg F., Kloas W. (2022). The aquaponic principle—It is all about coupling. Rev. Aquac..

[9] Bartelme R.P., Oyserman B.O., Blom J.E., Sepulveda-Villet O.J., Newton R.J. (2018). Stripping away the soil: Plant growth promoting microbiology opportunities in aquaponics. Front. Microbiol..

[10] Conijn J.G., Bindraban P.S., Schröder J.J., Jongschaap R.E.E. (2018). Can our global food system meet food demand within planetary boundaries?. Agric. Ecosyst. Environ..

[11] Wittebolle L., Marzorati M., Clement L., Balloi A., Daffonchio D., Heylen K., De Vos P., Verstraete W., Boon N. (2009). Initial Community Evenness Favours Functionality under Selective Stress. Nature.

[12] Goddek S., Espinal C.A., Delaide B., Jijakli M.H., Schmautz Z., Wuertz S., Keesman K.J. (2016). Navigating towards decoupled aquaponic systems: A system dynamics design approach. Water.

[13] Lobanov V., Monsees H., Latrille F., Pate G., Joyce A. (2026). Coupled vs. Decoupled Aquaponics: Evidence, Mechanisms, and Methodological Gaps in the Productivity Hypothesis. Aquacult. Eng..

[14] Tabassum T., Mahamud A.G.M.S.U., Acharjee T.K., Hassan R., Akter Snigdha T., Islam T., Alam R., Khoiam M.U., Akter F., Azad M.R. (2021). Probiotic supplementations improve growth, water quality, hematology, gut microbiota and intestinal morphology of Nile tilapia. Aquac. Rep..

[15] Monsees H., Suhl J., Paul M., Kloas W., Dannehl D., Würtz S. (2019). Lettuce (*Lactuca sativa*, variety Salanova) production in decoupled aquaponic systems: Same yield and similar quality as in conventional hydroponic systems but drastically reduced greenhouse gas emissions by saving inorganic fertilizer. PLoS ONE.

[16] Matysiak B., Kaniszewski S., Mieszczakowska-Frąc M. (2023). Growth and quality of leaf and romaine lettuce grown on a vertical farm in an aquaponics system: Results of farm research. Agriculture.

[17] Nadeem A., Shahzad H., Ahmed B., Muntean T., Waseem M., Tabassum A. (2022). Phytochemical Profiling of Antimicrobial and Potential Antioxidant Plant: *Nepeta cataria*. Front. Plant Sci..

[18] Li D.-D., Li J.-Y., Hu Z.-Q., Liu T.-X., Zhang S.-Z. (2022). Fall Armyworm Gut Bacterial Diversity Associated with Different Developmental Stages, Environmental Habitats, and Diets. Insects.

[19] Deng Q.Q., Luo X.Q., Li S.H., Li J.L., Wang P.D., Yuan Y., Yang Z.W., Li W.J. (2023). *Alsobacter* ponti sp. nov., a novel denitrification and sulfate reduction bacterium isolated from Pearl River sediment. Antonie van Leeuwenhoek.

[20] Huang Z., Wan R., Song X., Liu Y., Hallerman E., Dong D., Zhai J., Zhang H., Sun L. (2016). Metagenomic analysis shows diverse, distinct bacterial communities in biofilters among different marine recirculating aquaculture systems. Aquacult. Int..

[21] Wang B., Hu K., Li C., Zhang Y., Hu C., Liu Z., Ding J., Chen L., Zhang W., Fang J. (2024). Geographic Distribution of Bacterial Communities of Inland Waters in China. Environ. Res..

[22] Ghylin T., Garcia S., Moya F., Whitman W.B., Parshon S., Blom J., Dasgupta J., Durkin A.S., Korlach J., Lucchesi A. (2014). Comparative Single-Cell Genomics Reveals Potential Ecological Niches for the Freshwater acI Actinobacteria Lineage. ISME J..

[23] Kielak A.M., Barreto C.C., Kowalchuk G.A., van Veen J.A., Kuramae E.E. (2016). The Ecology of Acidobacteria: Moving beyond Genes and Genomes. Front. Microbiol..

[24] Wongkiew S., Park M.-R., Chandran K., Khanal S.K. (2018). Aquaponic Systems for Sustainable Resource Recovery: Linking Nitrogen Transformations to Microbial Communities. Environ. Sci. Technol..

[25] Philippot L., Raaijmakers J.M., Lemanceau P., van der Putten W.H. (2013). Going Back to the Roots: The Microbial Ecology of the Rhizosphere. Nat. Rev. Microbiol..

[26] Guyonnet J.P., Guillemet M., Dubost A., Simon L., Ortet P., Barakat M., Heulin T., Achouak W., Haichar F.Z. (2018). Plant Nutrient Resource Use Strategies Shape Active Rhizosphere Microbiota through Root Exudation. Front. Plant Sci..

[27] Coskun D., Britto D.T., Shi W., Kronzucker H.J. (2017). Nitrogen Transformations in Modern Agriculture and the Role of Biological Nitrification Inhibition. Nat. Plants.

[28] Wang H., Yang L., Yang Y., Jia L. (2026). Multifunctional Bimetallic MOF Engineering: Innovative Synthesis and Versatile Applications in Adsorption, Catalysis, Sensing, and Energy. Coord. Chem. Rev..

[29] Wang H., Guo X., Yang L. (2026). Advanced Metal–Organic Frameworks for Next-Generation Energy Storage and Conversion: From Nanoscale Design to Widespread Applications. Nano Res..

[30] Badri D.V., Vivanco J.M. (2009). Regulation and function of root exudates. Plant Cell Environ..

[31] Neumann G., Bott S., Ohler M.A., Mock H.-P., Lippmann R., Grosch R., Smalla K. (2014). Root exudation and root development of *Lactuca sativa* (*Lactuca sativa* L. cv. Tizian) as affected by different soils. Front. Microbiol..

[32] Skorić M., Šiler B., Lukić T., Stupar A. (2026). Heterogeneity of iridoid biosynthesis in catmints: Molecular background in a phylogenetic context. J. Integr. Plant Biol..

[33] Stringlis I.A., Yu K., Feussner K., de Jonge R., van Bentum S., van Verk M.C., Berendsen R.L., Bakker P.A.H.M., Feussner I., Pieterse C.M.J. (2018). MYB72-dependent coumarin exudation shapes root microbiome assembly to promote plant health. Proc. Natl. Acad. Sci. USA.

[34] Adiguzel A., Ozer H., Sokmen M., Gulluce M., Sokmen A., Kilic H., Sahin F., Baris O. (2009). Antimicrobial and Antioxidant Activity of the Essential Oil and Methanol Extract of *Nepeta cataria*. Pol. J. Microbiol..

[35] Kwak M.J., Kong H.G., Choi K., Kwon S.K., Song J.Y., Lee J., Lee P.A., Choi S.Y., Seo M., Lee S.W. (2018). Rhizosphere microbiome structure alters to enable wilt resistance in tomato. Nat. Biotechnol..

[36] Ruiz A., Scicchitano D., Palladino G., Nanetti E., Candela M., Furones D., Sanahuja I., Carbó R., Gisbert E., Andree K.B. (2023). Microbiome study of a coupled aquaponic system: Unveiling the independency of bacterial communities and their beneficial influences among different compartments. Sci. Rep..

[37] Lobanov V., Keesman K.J., Joyce A. (2022). Plants dictate root microbial composition in hydroponics and aquaponics. Front. Microbiol..

[38] Rakocy J.E., Tidwell J.H. (2012). Aquaponics: Integrating Fish and Plant Culture. Aquaculture Production Systems.

[39] Sheridan C., Depuydt P., De Ro M., Petit C., Van Gysegem E., Delaere P., Dixon M., Stasiak M., Aciksöz S.B., Frossard E. (2017). Microbial community dynamics and response to plant growth-promoting microorganisms in the rhizosphere of four common food crops cultivated in hydroponics. Microb. Ecol..

[40] Erozi B.S., Fitzsimmons K. (2017). Phosphorus dynamics modeling and mass balance in an aquaponics system. Agric. Syst..

[41] Rani S., Kumar P., Dahiya P., Maheshwari R., Dang A.S., Suneja P. (2022). Endophytism: A Multidimensional Approach to Plant–Prokaryotic Microbe Interaction. Front. Microbiol..

[42] Rakocy J., Shultz R.C., Bailey D.S., Thoman E.S. (2004). Aquaponic Production of Tilapia and Basil: Comparing a Batch and Staggered Cropping System. Acta Hortic..

[43] Schmautz Z., Graber A., Jaenicke S., Goesmann A., Junge R., Smits T.H.M. (2017). Microbial Diversity in Different Compartments of an Aquaponics System. Arch. Microbiol..

[44] Krastanova M., Sirakov I., Ivanova-Kirilova S., Yarkov D., Orozova P. (2022). Aquaponic Systems: Biological and Technological Parameters. Biotechnol. Biotechnol. Equip..

[45] Hahn M.W., Schmidt J., Taipale S.J., Doolittle W.F., Koll U. (2014). *Rhodoluna lacicola* gen. nov., sp. nov., a planktonic freshwater bacterium with streamlined genome. Int. J. Syst. Evol. Microbiol..

[46] Neuenschwander S.M., Ghai R., Pernthaler J., Salcher M.M. (2018). Microdiversification in Genome-Streamlined Ubiquitous Fresh water Actinobacteria. ISME J..

[47] Ogata Y., Watanabe K., Takemine S., Kaida K., Tanokura M., Suda W., Maresca J.A. (2022). Complete Genome Sequences of *Rhodoluna* sp. Strains KAS3 and KACHI23, Isolated from Lake and River Surface Water. Microbiol. Resour. Announc..

[48] McBride M.J. (2019). Bacteroidetes gliding motility and the type IX secretion system. Microbiol. Spectr..

[49] Yu S., Lu X., Lu H. (2025). Marine Microbial Biofilms on Diverse Abiotic Surfaces. Front. Mar. Sci..

[50] McBride M.J., Zhu Y. (2013). Gliding motility and Por secretion system genes are widespread among members of the phylum Bacteroidetes. J. Bacteriol..

[51] Hudson J., Egan S. (2022). Opportunistic Diseases in Marine Eukaryotes: Could Bacteroidota Be the Next Threat to Ocean Life?. Environ. Microbiol..

[52] Su L., Wu H., Yang M., Su Y., Wu S., Gao H., Li Y., Wang D., Lu Y., Zhang K. (2025). Purification Effect and Microbial Community Analysis of Aquaculture Wastewater Using High-Efficiency and Stable Biochemical System. Water.

[53] Khan S.T., Horiba Y., Yamamoto M., Hiraishi A. (2002). Members of the Family Comamonadaceae as Primary Poly(3-Hydroxybutyrate-co-3-Hydroxyvalerate)-Degrading Denitrifiers in Activated Sludge as Revealed by a Polyphasic Approach. Appl. Environ. Microbiol..

[54] Karanasios K.A., Vasiliadou I.A., Pavlou S., Vayenas D.V. (2010). Hydrogenotrophic Denitrification of Potable Water: A Review. J. Hazard. Mater..

